# FT-MIR-ATR Associated with Chemometrics Methods: A Preliminary Analysis of Deterioration State of Brazil Nut Oil

**DOI:** 10.3390/molecules28196878

**Published:** 2023-09-29

**Authors:** Braian Saimon Frota da Silva, Nelson Rosa Ferreira, Priscila Domingues Alamar, Thiago de Melo e Silva, Wandson Braamcamp de Souza Pinheiro, Lucely Nogueira dos Santos, Cláudio Nahum Alves

**Affiliations:** 1 Graduate Program in Chemistry, Federal University of Pará (PPGQ), Belém 66075-110, Brazil; thiagoms30@gmail.com (T.d.M.e.S.); wbraamcamp@ufpa.br (W.B.d.S.P.); nahum@ufpa.br (C.N.A.); 2 Faculty of Food Engineering, Institute of Technology, Federal University of Pará (UFPA), Belém 66075-110, Brazil; nelson.ufpa@gmail.com; 3 Laboratory of Biotechnological Processes (LABIOTEC), Graduate Program in Food Science and Technology (PPGCTA), Institute of Technology (ITEC), Federal University of Pará (UFPA), Belém 66075-110, Brazil; dra.alamar@gmail.com (P.D.A.); lucelynogueira@gmail.com (L.N.d.S.)

**Keywords:** Brazil nuts, acidity index, peroxide index, vegetable oil, FT-MIR-ATR, PCA, PLSR, economic sectors

## Abstract

Brazil nut oil is highly valued in the food, cosmetic, chemical, and pharmaceutical industries, as well as other sectors of the economy. This work aims to use the Fourier transform infrared (FTIR) technique associated with partial least squares regression (PLSR) and principal component analysis (PCA) to demonstrate that these methods can be used in a prior and rapid analysis in quality control. Natural oils were extracted and stored for chemical analysis. PCA presented two groups regarding the state of degradation, subdivided into super-degraded and partially degraded groups in 99.88% of the explained variance. The applied PLS reported an acidity index (AI) prediction model with root mean square error of calibration (RMSEC) = 1.8564, root mean square error of cross-validation (REMSECV) = 4.2641, root mean square error of prediction (RMSEP) = 2.1491, R^2^_cal_ (calibration correlation coefficient) equal to 0.9679, R^2^_val_ (validation correlation coefficient) equal to 0.8474, and R^2^_pred_ (prediction correlation coefficient) equal to 0, 8468. The peroxide index (PI) prediction model showed RMSEC = 0.0005, REMSECV = 0.0016, RMSEP = 0.00079, calibration R^2^ equal to 0.9670, cross-validation R^2^ equal to 0.7149, and R^2^ of prediction equal to 0.9099. The physical–chemical analyses identified that five samples fit in the food sector and the others fit in other sectors of the economy. In this way, the preliminary monitoring of the state of degradation was reported, and the prediction models of the peroxide and acidity indexes in Brazil nut oil for quality control were determined.

## 1. Introduction

The Brazil nut (*Bertholletia excelsa*) is a seed belonging to the Amazon biome, recognized worldwide for its high nutritional value. For this reason, it can be applied in food preparation, pharmaceuticals, and cosmetics. In addition, Brazil nut harvesting is an important source of income for local communities in the Amazon. Promoting sustainable harvesting is crucial to preserving the forest and economically supporting these communities while ensuring the continuity of this valuable natural resource [1,2,3,4].

Brazil nut oil extracted via mechanical pressing has an average yield of 60.8% [2]. The composition includes saturated fatty acids, including palmitic acid (~15%) and stearic acid (~10%), as well as monounsaturated fatty acids, such as oleic acid (~40%) and polyunsaturated fatty acids (omega 3 and omega 6) but with a greater amount of linoleic acid (~34%) [5]. These compounds are essential in human health, being fundamental components of cell membranes, regulating inflammatory processes and blood clotting, and acting as precursors of hormones, preventing cardiovascular and neurodegenerative diseases and metabolic syndromes [6,7,8].

Brazil nut oil is enriched with a variety of nutrients, such as tocopherols; phytosterols; flavonoids; essential minerals, such as magnesium, calcium, selenium, zinc, potassium, phosphorus, and copper; and vitamins, including niacin, pyridoxine, and thiamine. This combination of components contributes to the high quality and nutritional benefits of Brazil nut oil, making it a healthy and versatile choice in many applications, from food to skin care products and supplements [9,10]. In this way, Brazil nut oil contains several bioactives, such as antioxidants that regulate the immune system, in addition to mitigating the incidence of cardiovascular pathologies and excluding risk factors such as oxidative stress, inflammation, high cholesterol, and diabetes [11,12,13,14].

This way, the quality of Brazil nut oil for edible and other purposes is measured through experimental analyses in terms of acidity, peroxide, and saponification; according to Agência de Vigilância Sanitária (ANVISA) [15], methods established in the scientific literature, examine factors such as color, density, pH, viscosity, refractive index, among others [16,17,18,19].

The acidity index, when related to the oleic acid content, is one of the fundamental parameters in assessing the quality of oils. For example, low-acid vegetable oils can help prevent cardiovascular diseases, such as atherosclerosis and hypertension, as well as metabolic syndromes, including type 2 diabetes and obesity. In addition, they play a role in preventing oncological conditions, such as breast, prostate, and colon cancer. These oils promote homeostasis in the human body, contributing to a healthy balance and helping to reduce the risk of these chronic diseases [20,21].

Another fundamental quality criterion is the peroxide number, which evaluates the primary oxidation compounds, including peroxides and hydroperoxides. These components trigger reactions that can result in the formation of secondary compounds, exerting a significant influence on characteristics such as color, flavor, aroma, viscosity, and other parameters indicative of high quality oil. Maintaining low levels of peroxide numbers is essential to preserving the integrity and desired properties of the oil, thus ensuring its suitability for a variety of applications and customer satisfaction [21,22,23,24].

However, it is essential to note that traditional chemical analyses can be time consuming and require significant effort, even when the procedures themselves are relatively simple. An innovative approach has been the application of mid-infrared spectroscopy, combined with chemometric methods. This approach offers a more efficient alternative to determine quality parameters and identify possible adulterations in vegetable oils. Mid-infrared spectroscopy allows a quick and accurate analysis of the chemical composition of oil samples, while chemometric methods aid in data interpretation, making the process more agile and reliable. This combination of techniques is becoming increasingly valuable in the food industry and in ensuring the quality of vegetable oils [25,26,27].

A chemometric analysis applicable in this work is the principal component analysis (PCA) method, which allows the graphic contemplation of an entire dataset, especially when the number of samples and variables is high [28,29]. In one study, it was possible to detect the adulteration of sesame oil by applying PCA [30]. In another article, this statistical method determined a conceptual view of the similarities and discrepancies between samples of different oils extracted in the Amazon as a function of several chemical variables [31].

Another relevant chemometric analysis in this research is the partial least squares regression (PLSR). This supervised regression method considers the known characteristics of a certain process, compound, or natural phenomenon [32,33]. When applying this method, spectra are provided in the mid-infrared region representing the “matrix X”, which seeks a direct relationship with the variables of interest (IV) represented by the “matrix Y” (in this case, AI and PI) [34,35,36].

Therefore, this work aims to evaluate the deterioration of oils extracted from Brazil nuts using FT-MIR-ATR spectroscopy techniques and chemometric methods (PCA and PLSR) in order to identify patterns groupings between samples, in addition to developing predictive models to assist in the preliminary quality control of these oils. These models have the potential to provide valuable insights into the quality of products, enabling informed decision making in the industry and other related sectors.

## 2. Results and Discussion

### 2.1. Experimental Analyses

#### 2.1.1. Acidity Index (AI)

**Figure 1:**
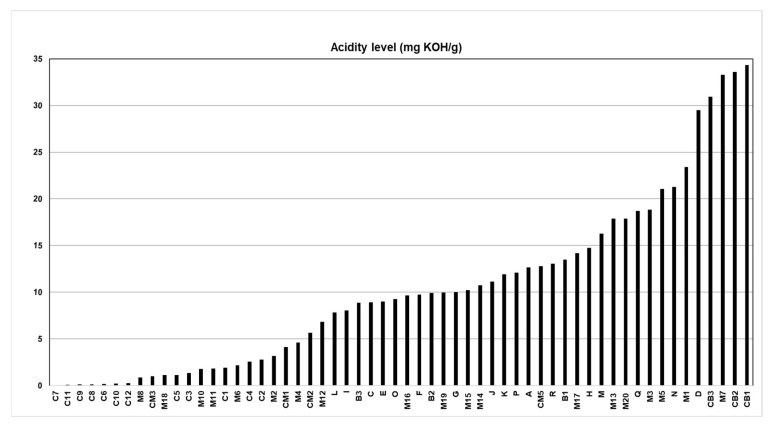
Acidity index of Brazil nut oil.

Figure 1 shows the results of the acidity index (AI) of the 58 batches of Brazil nut oil. The lowest AI was equivalent to 0.05 mg of KOH/g for batch C7, and the highest AI was 34.32 mg of KOH/g for batch CB1.

Among the samples analyzed, only twenty samples of Brazil nut oil meet ANVISA’s technical regulations, resolution n° 270, for vegetable oils, fats and creams. [13]. These AI values were less than 4 mg KOH/g, which approves batches C7 to CM1 for edible purposes. In a study by Marinho et al. [37], silk fibroin nanoparticles with esters obtained from Brazil nut oil with low acidity indices promoted potential larvicidal activity and oviposition deterrence against Aedes aegypti. In several studies, Brazil nut oils with a low acidity index have already been used to treat depression in children and adolescents, preventing the non-degeneration of nervous tissue, among other cardiovascular pathologies that can be avoided [38,39].

Another 38 samples were above the stipulated by law for edible vegetable oils, which can be used in the production of cosmetics when refined and then reused in other economic activities [18,19,40]. Rincón, Cadavid, and Orjuela [41] evaluated the retention potential of used cooking oils as oleochemical feedstock for urban biorefineries in Colombia, and this research reported that graded oils with high acidity could be purified and reused and returned to the consumer market with high added value in various byproducts. Aghel et al. [42] conducted a study on the production of biodiesel from waste cooking oil of high acidity using the magnesium oxide nanocatalyst doped with graphene oxide for transesterification in a microreactor for refining and the subsequent reuse of the oil vegetable.

The high acidity indexes indicate that the oil samples may have been conditioned in an environment with inappropriate temperatures and excessive light, slightly oxidizing the oil without adding antioxidants [22,38,43].

#### 2.1.2. Peroxide Index (PI)

**Figure 2:**
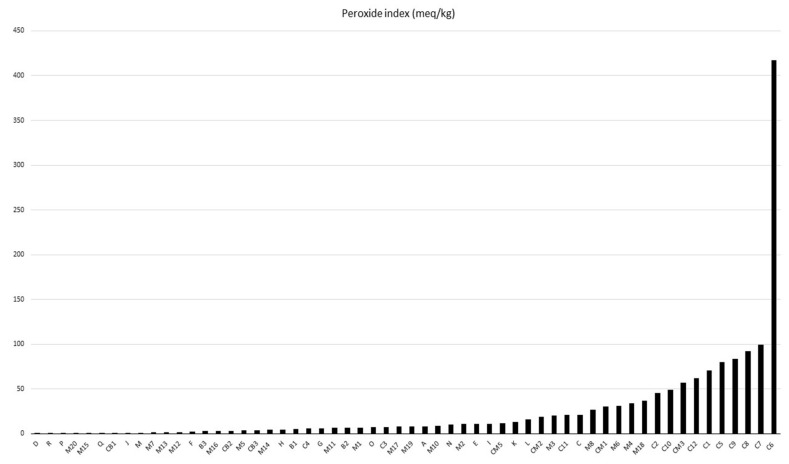
Peroxide index of Brazil nut oil.

Among the analyzed samples, only 38 complied with Agencia de Vigilância Sanitária, Resolution n° 270, the technical regulations for vegetable oils, fats, and creams [13]. In Figure 2, batches D to K present up to 15 meq O_2_/kg values. Only batches C3, C4, M10, M11, and M12 can be sent to the food sector, as they comply with the legislation in terms of AI and PI parameters. In a study by Kharbach, Alaoui, and Taabouz [44], various processed food products were analyzed. They found a strong correlation between the peroxide and acidity indexes and the sensory acceptability of the products. This highlights the importance of these indices not only for food safety but also for consumer satisfaction.

According to Cardoso et al. [45], high levels of peroxides in degraded vegetable oils above the limits stipulated by law can be reused in the production of bactericides. Furthermore, they can be used as soap and even in the production of biodiesel when properly refined. [38,46,47]. In this way, the sustainable management of these deteriorated vegetable oils is necessary because when they come into direct contact with the environment, they cause an environmental imbalance in aquatic and terrestrial ecosystems [48,49].

#### 2.1.3. FT-MIR Spectroscopy

**Figure 3:**
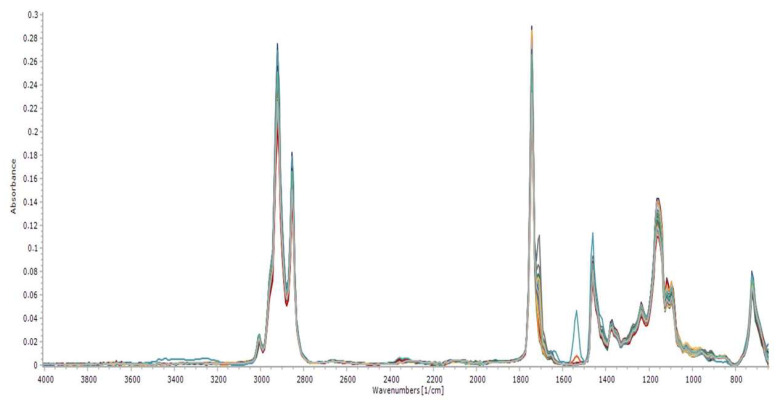
MIR spectra of Brazil nut vegetable oil.

Spectral absorbance data were concatenated into 451 wavenumber ranges in the mid-infrared (MIR) ranges, between 650 cm^−1^ to 4003 cm^−1^, from 58 Brazil nut samples, as shown in Figure 3.

An elongation band was observed in the C=O of methyl esters at 1743 cm^−1^, as well as elongation bands of C-O at 1170 cm^−1^, 1195 cm^−1^, and 1246 cm^−1^ and a weak signal at 1654 cm^−1^ due to the frequency stretching of C=C. Strong and sharp signals at 2854 cm^−1^ and 2926 cm^−1^ were due to the stretching frequencies of C-H. The absorbance at 3005 cm^−1^ indicated the frequency of the elongation of =C-H. An absorption band at 733 cm^−1^ of CH_2_ was noticed.

The superimposition of the spectra of Brazil nut oil showed a certain number of specific wave vibrations contained in oleic acid, which has relevant levels of concentration, being a reference for the calculation of the acidity index and the consolidation of the quality and destination of this matrix in several economy sectors [50,51]. According to Perez-Nakai et al. [2], this oil mainly comprises oleic and linoleic fatty acids. This unique lipid composition gives the Brazil nut significant potential for several applications, especially biopolymers.

### 2.2. Chemometrics

#### 2.2.1. PCA Modeling Results

**Figure 4:**
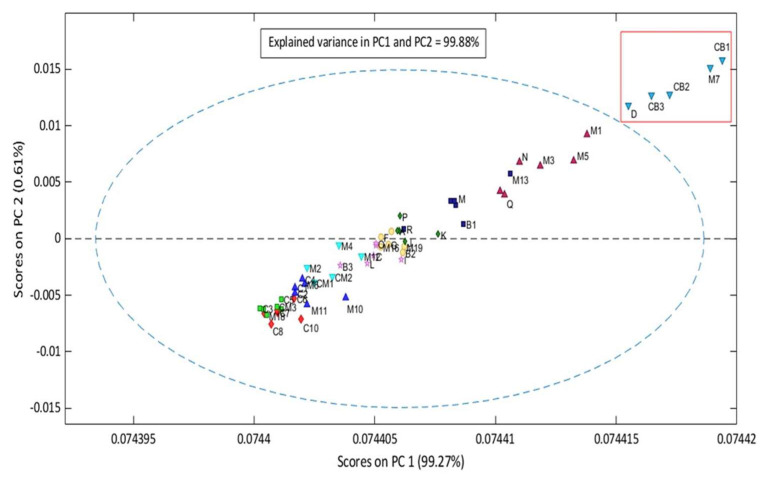
Grouping between lots of Brazil nuts.

To discriminate similarities and discrepancies between batches in different groups, principal component analysis (PCA) was applied as a function of mid-infrared spectroscopy (Figure 4). The first two principal components (PCs) explain 99.88% of the variance. However, the CM5 sample was removed due to its high residue and is considered an outlier. This way, the model was designed for 451 wavenumber ranges and 57 samples. In a quick analysis, it can be observed through principal component analysis that the most degraded samples presented high scores. Thus, in terms of the possibility of inserting very degraded unknown samples, the model tends to position them in the region of the most accentuated scores.

In a study carried out by De Menezes et al. [52], the principal component analysis associated with the FT-MIR-ATR was used to investigate the alteration of biodiesel that can be degraded when exposed to certain weather conditions such as air, light, temperature, and humidity, consequently changing its quality parameters, among them the acidity index. Thus, the model proposed a total variation of 75% of the proposed environmental conditions.

According to Herculano et al. [29], the oxidative stability of a set of eighteen samples of edible oils (avocado, peanut, safflower, sesame, brown linseed, macadamia, primrose, pumpkin seed, soybean, cottonseed, rice, chia, sunflower, golden linseed, piece of walnut, canola, grape seed, and Brazil nut) were grouped into three classes through the application of PCA combined with FT-MIR, which registered 92% of total variance. When fatty acids are oxidized, double bonds are broken, and oxidative compounds such as peroxides and aldehydes are formed, forming free fatty acids and increasing the acid number of vegetable oils [53,54,55].

**Figure 5:**
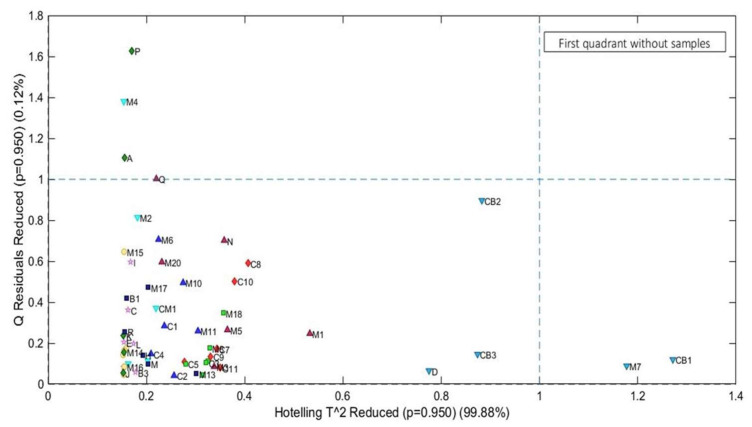
Hotelling’s T^2^ test.

The PCs with the highest and lowest scores were linearly conditioned in two different spaces, with the first group (inside the red rectangle) comprising batches D, CB3, CB2, M7, and CB, ranging from 29.52 to 34.31 mg of KOH/g. These batches had the highest scores in the model, which categorized them into the group of super-degraded samples. The second group of partially degraded samples is within the ellipse, within an AI range between 0.05 mg KOH/g and 23.52 mg KOH/g. Finally, to consolidate the quality of the model, Hotelling’s T^2^ test was applied (Figure 5), which evaluates the screening of outliers based on the weighted sum of squared scores and the sum of squared residuals, where two samples (M7 and CB1) showed a strong influence on the PCA model and four samples (Q, A, M4, and P) had high residuals. However, none of the samples overlapped in the first quadrant, which characterizes it as an inefficient model.

Vegetable oils, when not stored correctly, can experience an increase in acidity when the temperature increases [56]. The increase in temperature can influence the deterioration of oils, with a decrease in absorbance at peaks close to 1655 cm^−1^, 1401 cm^−1^, and 1119 cm^−1^ and an increase at 1635 cm^−1^ and 1418 cm^−1^ in a model stipulated by the PCA with an accumulated variance of 95.23% [57].

In the study of Sousa et al. [58], the oxidative status was evaluated by the AI range, the spectral matrix was preprocessed by the multiplicative scattering correction and the standard normal variation with 98% of the explained variance, where the alterations were observed in the period of 5 days in the following regions: 1000 cm^−1^ to 1800 cm^−1^ and 1228 cm^−1^ to 1163 cm^−1^, with peaks at 1739 cm^−1^ and 3330 cm^−1^. This model was also consolidated from 30 samples submitted to artificial weathering, intensifying the increase in temperature of the babassu oil. In this study, the Brazil nut oil samples were processed at room temperature without forcing extreme conditions. This highlights the real conditions in the extraction process until the final destination, where the oil is made available to a specific economic sector.

#### 2.2.2. Results of Calibration and Prediction through PLSR

The partial least squares regression method was applied to predict two variables of interest, AI and PI, in 58 natural Brazil nut oils samples. Table 1 presents the variables of interest (IV), the number of bands (N) of the preprocessing MIR spectra, RMSEC, REMSECV, RMSEP, the number of latent variables (LV), calibration R^2^ (R^2^_cal_), cross-validation R^2^ (R^2^_val_), and the R^2^ of prediction (R^2^_pred_).

**Figure 6:**
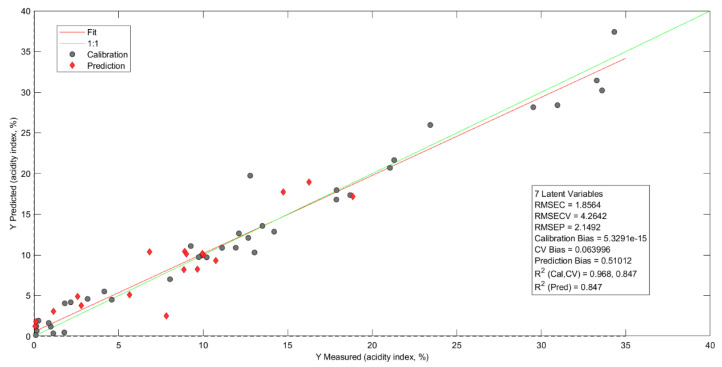
AI prediction model.

Figure 6 reports the prediction model of AI, which was used in the MC, MSC, and 1D preprocessing. This procedure minimizes the dispersions between the sample spectra and the baseline, corrects for critical points in the calibration, and determines whether each point is a local maximum, local minimum, or zero slope point caused by particles in the oil [59,60,61,62].

In a study by Fetter et al. [63], a quadratic cross-validation error was observed, with an average value of 1.6895 with 13 latent variables in the two models proposed to predict AI in frying oils. Pre-processing in the first model employed data normalization, 1D, and MC. In the second model, normalization, MSC, and MC were used for 13 latent variables. In this study, the AI prediction reported a calibration mean square error of 1.8564, R^2^_cal_ = 0.9679, RMSEP = 2.1491, R^2^_val_ = 0.8474, and R^2^_pred_ = 0.8468, and the cross-validation mean square error was 4.2641 with only seven latent variables. This indicated a reduction in the dimensionality of the data, which optimized the prediction of the variable of interest [64,65,66].

**Figure 7:**
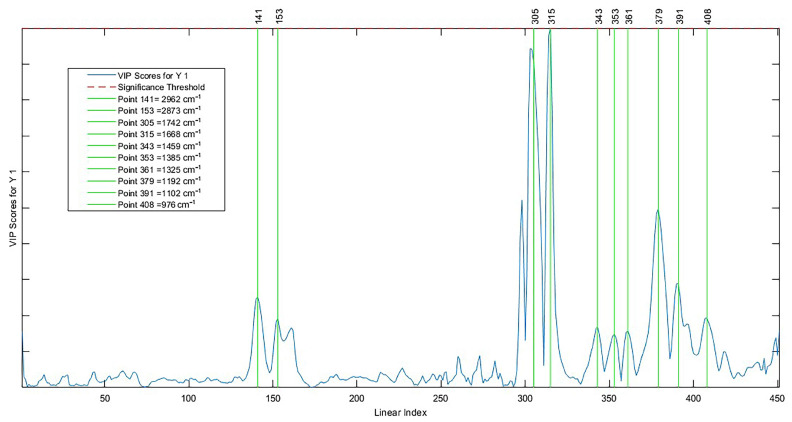
Important variables in the projection of the AI model.

The wave numbers (Figure 7) that most contributed to the AI predictive model were 2962 cm^−1^, 2873 cm^−1^, 1742 cm^−1^, 1668 cm^−1^, 1459 cm^−1^, 1385 cm^−1^, 1325 cm^−1^, 1192 cm^−1^, 1102 cm^−1^, and 976 cm^−1^. Such discriminatory variables were evidenced through the importance of the variable in the projection (VIP); this represents the weighted sum of squares of the PLS weights as a function of the amount of explained variance of the dependent variable in each component. These wave numbers cover the fingerprint region and the C-H elongation bands, which in vegetable oils denote a high level of unsaturated fatty acids, such as oleic acid and linoleic acid, and together make up more than 70% of Brazil nut oil [67,68].

**Figure 8:**
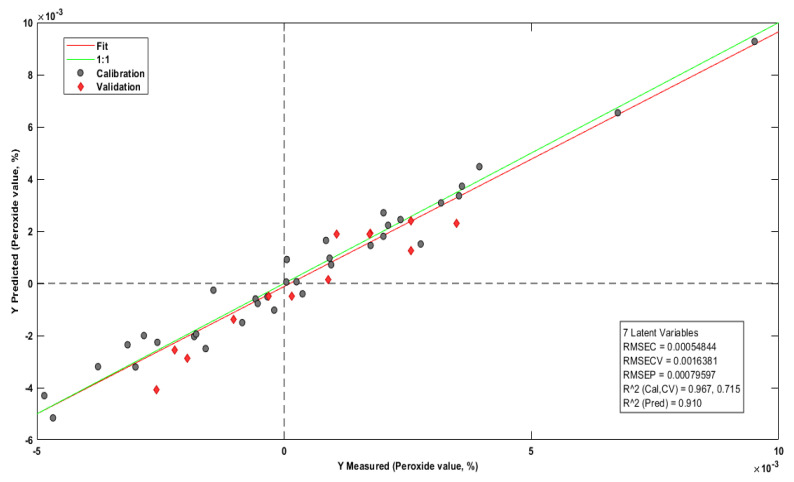
PI prediction model.

Figure 8 reports the prediction model for the peroxide index. Five samples were withdrawn for presenting high residues, and the column representing the wave number 4003 cm^−1^ was excluded for having a high negative influence on the model.

In research on the prediction of quality parameters in natural vegetable oils carried out by [69], the PI prediction model, which used Raman spectroscopy in ranges from 430 cm^−1^ to 2700 cm^−1^, was also created with seven latent variables with RMSEC = 0.85, RMSECV = 1.47, R^2^cal = 0.91, RMSEP = 0.95, and R^2^pred = 0.86. In this PI prediction model, the squared errors of calibration (RMSEC = 0.0005) and cross-validation (RMSECV = 0.0016) were significantly lower.

According to Souza et al. [58], the related predictions of the peroxide content in 30 samples of babassu oil (Attalea speciosa) reported the coefficient of determination (R^2^) equal to 0.94, RMSECV equal to 4.7, and RMSEP equal to 7.85, in mid-infrared between ranges of 1000 cm^−1^ to 1800 cm^−1^. Many calibrations, cross-validations, and prediction errors were minor in this PI prediction model for Brazil nut oil. In addition, the correlation coefficients in the calibration and validation were higher, indicating greater robustness in the model of this study.

**Figure 9:**
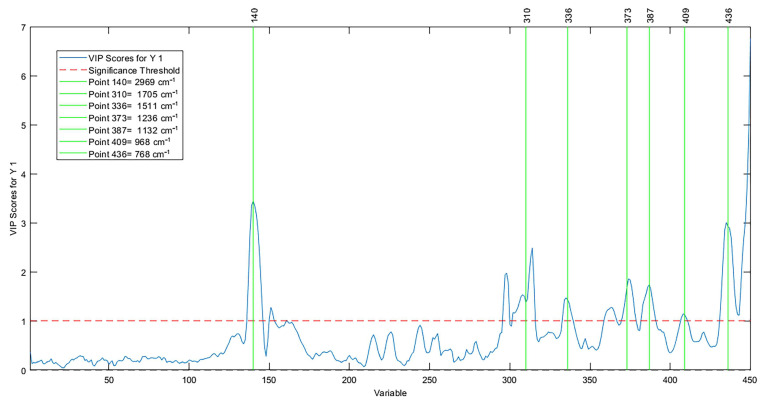
Important variables in the projection in the PI model.

The most contributed abscissa variables in the spectrum were 2969 cm^−1^, 1705 cm^−1^, 1511 cm^−1^, 2337 cm^−1^, 1236 cm^−1^, 1132 cm^−1^, 968 cm^−1^, and 768 cm^−1^. A survey by Okere et al. [70] proved that the tracks between 4000 cm^−1^ to 1030 cm^−1^ change, revealing the oxidation state in vegetable oils and, consequently, the quantification of high PI values in prediction models. Figure 9 reports the selection of discriminatory variables through the importance of the variable in the projection.

In this context, the MIR spectra of natural Brazil nut oils associated with PLSR can weigh AI and PI predictive models. Thus, inserting new spectral data from unknown samples of this plant matrix enables the rapid prediction of such parameters and mitigates the time needed for results and the expensive use of chemical reagents used in physical–chemical analyses. Therefore, it can be applied as a sustainable tool to secondary sectors of the economy. However, for the concatenation of such predictive models, it is necessary to pay attention to the quality of the plant matrix of interest, the large number of samples for robust modeling, the natural oil extraction process, and the obtaining of bench data from the physical–chemical analyses, which were faithfully executed, in order not to negatively influence the multivariate calibration model [71,72,73,74,75,76].

## 3. Materials and Methods

### 3.1. Obtaining Samples

The study used a total of 58 batches of Brazil nuts, visually super-degraded (low quality) and partially degraded samples (with qualities ranging from good to excellent), donated by a company located in Belém do Pará, Brazil, at the geographic coordinates 1°27′21″ S, 48°30′14″ W. After collection, the seeds were subjected to a drying process in an oven (Inova 220v model, Votorantim, São Paulo, Brazil) at a temperature of 45 °C for six hours. Next, in the drying process, the oils from the samples were extracted through mechanical press (ERT 60, from Scoot Tech, Vinhedo, São Paulo, Brazil) and stored correctly in opaque plastic containers to preserve their characteristics and quality over time.

### 3.2. Experimental Analyses

#### 3.2.1. Acidity Index

The acidity index is the result of the number of milligrams of KOH needed to neutralize 1 g of the natural oil sample; according to the American Oil Chemists’ Society—AOCS, Cd 3d-63 [77], the free fatty acid content can be calculated in triplicate according to the Equation (1):

(1) $$AI\;\left(mg\;of\frac{KOH}{g}\right)=\frac{\left(A-B\right)\times N\times 56.1056\times f}{w}$$

where *B* = blank volume; *A* = sample volume; *N* = KOH normality; 56.1056 = molar mass of KOH; *f* = KOH correction factor equal to 0.9935; and *w* = sample mass in grams.

#### 3.2.2. Peroxide Index

According to AOCS, Cd 8-53 [78], the peroxide index (*PI*) is determined as the amount of oxygen peroxide per kilogram of oil, where the amount of peroxide is obtained in milliequivalents (meq), in triplicate, through the following Equation (2):

(2) $$PI\;\left(mg\;O₂/Kg\right)=\frac{\left(A-B\right)\times N\times f\times 1000}{w}$$

Such that *A* = amount in mL of 0.1 N sodium thiosulphate solution used in the titration; *B* = amount in mL of 0.1 N sodium thiosulphate solution used in the blank titration; *N* = normality of the sodium thiosulphate solution; *f* = sodium thiosulphate solution correction factor equal to 0.9950; and *w* = quantity in grams of the sample.

#### 3.2.3. FT-MIR Spectroscopy

Mid-infrared spectroscopy (MIR) analyses were performed in the range of 650 cm^−1^ to 4000 cm^−1^ using the Agilent Cary 630 FT-MIR spectrometer (Santa Clara, CA, USA) with an attenuated total reflection (ATR) module and zinc selenide crystal. The resolution was 16 cm^−1^ with 32 scans. The volume of each oil sample was 20 µL.

### 3.3. Statistical Treatment

#### 3.3.1. Principal Components Analysis (PCA)

The PCA was applied to explore a reduced dimension of the media-centered spectral data obtained from the FT-MIR-ATR, transforming the original time series data into a smaller set of linear mutations with different patterns of variation in order to monitor oil discrimination in different degradation ranges. This way, the score graph and the weight graph are generated using principal components (58 vegetable oil samples and 451 wavenumber ranges) by Matlab R2021b software (Mathworks, Natick, MA, USA) and PLS_Toolbox 9.2 (Eigenvector Research Inc., Wenatchee, WA, USA).

#### 3.3.2. Partial Least Squares Regression (PLSR)

Mean centralization (MC), multiplicative dispersion correction (MSC), and first derivative (1D) were applied with preprocessing using the Savitzky–Golay algorithm with a 15-point smoothing window and second-order polynomials. Thus, the calibration spectra were subjected to partial least squares regression (PLSR) with “continuous block” cross-validation used to determine the number of latent variables (LV). The original dataset was divided into two subsets selected via the Kennard–Stone algorithm: the calibration set contained 2/3 of the samples and the validation set contained 1/3. The performance of the PLSR calibration models was evaluated using the coefficient of determination (R^2^), mean squared error of calibration (RMSEC), mean squared error of cross-validation (RMSECV), and mean squared error of prediction (RMSEP). The modeling was concatenated using the PLS toolbox 9.2 (Eigenvector Research Inc., Wenatchee, WA, USA) in the test version of Matlab R2021b software (Mathworks, Natick, MA, USA).

## 4. Conclusions

We performed AI and IP chemical analyses and found that only five Brazil nut batches met the necessary standards for the food industry. However, the 53 batches that did not comply with the regulations could still be used in other economic sectors with proper processing based on the user’s needs. We used principal components analysis associated with mid-infrared spectroscopy to identify groups of super-deteriorated and partially deteriorated oils. In contrast, FT-MIR-ATR combined with PLSR provided a reliable estimate of AI and PI prediction, showing low RMSEC, RMSECV, and RMSEP and moderate to strong correction coefficients in calibration, cross-validation, and prediction. These models can incorporate new external spectral data, thus expanding the quality control capacity for versatile matrices applicable in different economic sectors. Although the models are currently operational, they can be improved and optimized in future studies by refining algorithms, including more training data, and exploring other spectral variables that can improve prediction accuracy.

## Figures and Tables

**Table 1:** PLS results for AI and PI prediction.

IV	N	LV	RMSEC	RMSECV	R^2^_cal_	RMSEP	R^2^_val_	R^2^_pred_
AI	451	7	1.8564	4.2641	0.9679	2.1491	0.8474	0.8468
PI	450	7	0.0005	0.0016	0.9670	0.00079	0.7149	0.9099

## Data Availability

Not applicable.
